# Experimental Pain Sensitivity and Parental Pain Catastrophizing

**DOI:** 10.3390/children11121528

**Published:** 2024-12-17

**Authors:** Gourav Banerjee, Joel Brown, Alana McMichael, Arbi Ben Abdallah, Sarah Buday, Deanna M. Barch, Thomas Baranski, Simon Haroutounian, Jacob AuBuchon, Hadas Nahman-Averbuch

**Affiliations:** 1 Washington University Pain Center, Department of Anesthesiology, Washington University School of Medicine, St. Louis, MO 63110, USA; gourav@wustl.edu (G.B.); brownjoel@wustl.edu (J.B.); amcmich@wustl.edu (A.M.); aba@wustl.edu (A.B.A.); sbuday@wustl.edu (S.B.); sharout@wustl.edu (S.H.); jdaubuchon@wustl.edu (J.A.); 2 Division of Clinical and Translational Research, Department of Anesthesiology, Washington University School of Medicine, St. Louis, MO 63110, USA; 3 Department of Psychological and Brain Sciences, Washington University School of Medicine, St. Louis, MO 63110, USA; dbarch@wustl.edu; 4 Division of Endocrinology, Metabolism and Lipid Research, Washington University School of Medicine, St. Louis, MO 63110, USA; baranski@wustl.edu

**Keywords:** pain catastrophizing, parent–child relations, quantitative sensory testing, parental worrying, pain modulation

## Abstract

Background/Objectives: Variability in biopsychosocial factors can explain the interindividual variability in pain. One factor that can impact pain is the pain catastrophizing level. Interestingly, parental pain catastrophizing is related to the severity of the clinical pain of their children. This study explored whether parental pain catastrophizing is also associated with their children’s experimental pain sensitivity. Methods: Forty-five healthy girls (mean age 12.07 ± 1.47 years) and one of their parents participated in this study. Parents completed the Pain Catastrophizing Scale (PCS) about their child’s pain (PCS-Parent_child_) as well as their pain (PCS-Parent). Children completed the PCS about their pain (PCS-Child) and the Pubertal Developmental Scale (PDS). Children underwent psychophysical tests, including paradigms of temporal summation, heat- and pressure-conditioned pain modulation, offset analgesia, and cold pain tolerance. Correlations and regression models were conducted to assess the relationships between parental pain catastrophizing scales (separately for PCS-Parent_child_ and PCS-Parent) and experimental pain sensitivity with and without controlling for PCS-Child and PDS. T-tests were used to compare pain sensitivity between participants with vs. without a family history of psychiatric disorder. Results: No significant relationships were found between the experimental pain sensitivity measures and either PCS-Parent_child_ or PCS-Parent with and without controlling for PCS-Child and PDS. No differences were found in experimental pain sensitivity between participants with and without a family history of psychiatric disorder. Conclusions: Parental pain catastrophizing may contribute minimally to the individual variability in experimental pain sensitivity of healthy adolescent girls.

## 1. Introduction

There is considerable individual variability in the sensitivity to experimental pain, which is influenced by various biopsychosocial factors [1,2]. In the context of pain variability in childhood and adolescence, one area that has received limited attention is the impact of parental psychological factors on their child’s pain sensitivity. In one study, parental anxiety was related to their child’s experimental pain sensitivity. This relationship was mediated via the effect of parental anxiety on the child’s anxiety and was observed only in girls [3]. In another study involving the manipulation of parental behavior, exaggerated parental pain expression was related to higher levels of anxiety and greater experimental pain intensity in their children, with girls reporting higher pain intensity than boys [4]. However, the role of parental pain catastrophizing on experimental pain has not been examined yet.

Pain catastrophizing refers to a maladaptive cognitive–emotional response to actual or anticipated pain, characterized by the exaggeration of perceived pain-related threats, persistent rumination on past pain experiences, and an inability to cope effectively with pain [5]. A positive relationship between pain catastrophizing and pain ratings, both clinical and experimentally induced, has been found, and patients with chronic pain typically exhibit higher levels of pain catastrophizing compared to healthy controls [5,6,7,8,9,10,11,12,13,14]. Pain catastrophizing is strongly related to pain conditions and serves as a robust predictor of chronic pain outcomes, including musculoskeletal pain and headaches [5,6,7,8,9]. A bidirectional and reinforcing relationship may exist between pain catastrophizing and pain, where one exacerbates the other [8,9,15,16,17]. For instance, experimental manipulations that increase pain catastrophizing have been shown to heighten pain perception in participants with and without chronic pain [16,18].

While the precise mechanisms remain unclear, it is theorized that pain catastrophizing exerts a negative influence on endogenous pain modulatory mechanisms, leading to greater pain sensitivity [19,20]. Studies have demonstrated a link between higher pain catastrophizing, greater experimental pain ratings, and reduced inhibitory pain modulation capabilities in adults, both with and without chronic pain [10,11,19,20,21,22]. Similarly, the experience of unresolved and intense pain may catalyze rumination (e.g., concerns about worsening pain over time leading to future disability), thereby contributing to an increase in pain catastrophizing [23,24].

The effect of parental catastrophizing on children’s pain has been less studied, but it is suggested that higher levels of parental pain catastrophizing are related to higher acute and chronic pain severity in their children [25,26]. Parental catastrophizing can lead to parents, verbally or non-verbally, expressing biased information about pain to their children. This may influence children’s beliefs and perceptions about pain, affecting their sensitivity to it [27,28,29,30,31,32]. For instance, parents with greater parental pain catastrophizing are more likely to limit their child’s participation in potentially painful activities [27], and these protective parental responses are linked to higher pain sensitivity in children with chronic pain [28].

Since parental pain catastrophizing is related to clinical pain severity in adolescents with acute or chronic pain conditions, we aimed to study if these relationships would be replicated in healthy adolescents. Thus, the aim of this study was to assess the relationships between parental pain catastrophizing and experimental pain sensitivity in healthy adolescents. We hypothesized higher parental pain catastrophizing scores would be correlated with increased experimental pain sensitivity and decreased pain modulation capabilities in healthy girls.

## 2. Materials and Methods

### 2.1. Participants

Healthy girl participants were recruited for this study. Recruitment methods included distributing flyers through the research participant registry of Washington University School of Medicine; *peachjar*, an online platform for sending flyers to parents via their children’s schools and community groups; and word of mouth. Inclusion criteria included the following: (1) age between 9 and 16 years; (2) not pregnant or breastfeeding; (3) with no diagnosis of chronic pain, psychiatric or neurological disorders, or disorders associated with pubertal maturation; (4) not using medications on a regular basis that may affect pain sensitivity (e.g., opioids, antidepressants); (5) able to comprehend simple instructions and communicate in English. Participants were asked not to take any pain or over-the-counter medications 24 h before the study visit. They were also advised to avoid engaging in activities, such as intense workouts, that could potentially lead to persistent pain or soreness in the days leading up to the study visit. Each participant received a compensation of USD 85 for participating in the study visit.

### 2.2. Experimental Procedure

Before the study participation, written informed consent and assent were obtained from all participants and one of their parents or guardians. Each child participant, along with one of their parents, completed questionnaires to capture socio-demographic, health, behavioral, and psychological information, including a familial history of diagnosed psychiatric disorders among first-degree relatives. The experimental pain sensitivity of the child participants was assessed while the parent remained in the waiting room located outside of the testing room. Participants were first familiarized with the experimental procedures and the pain rating scale. Pressure pain thresholds, pain modulation tests of temporal summation, conditioned pain modulation using pressure and heat modalities, offset analgesia, and cold pain tolerance were assessed. The study visit lasted about two and a half hours.

#### 2.2.1. Questionnaires

##### Pain Catastrophizing Scale (PCS)

Parents completed the PCS in relation to their own pain (PCS-Parent) [33] as well as in relation to their child’s pain (PCS-Parent_child_) [34]. Participants completed the child-adapted version of the PCS in relation to their own pain (PCS-Child) [35]. These PCS versions are validated and reliable tools for assessing catastrophic thinking about pain in both adults and children. Parents and children were asked to reflect on past painful experiences and rate their level of agreement on a Likert scale, ranging from 0 (experienced ‘not at all’) to 4 (experienced ‘all the time’ or ‘extremely’), for each of 13 thoughts or feelings associated with pain. The parental and child PCS versions contained similar questions, with minor variations in phrasing and or wording, for example, ‘When I’m in pain, I anxiously want the pain to go away’ (PCS-Parent) vs. ‘When my child is in pain, I want the pain to go away’ (PCS-Parent_child_) vs. ‘When I’m in pain, I want the pain to go away’ (PCS-Child). The PCS total score ranges from 0 to 52 and is calculated by summing the 13-item responses; higher PCS scores indicate greater pain-related catastrophizing.

##### Pubertal Developmental Scale (PDS)

The PDS is a valid and reliable instrument for assessing pubertal development [36]. During puberty, there are changes in experimental pain sensitivity [37,38]; thus, in order to ensure that individual differences in experimental pain sensitivity in adolescents are not due to differences in pubertal status, the PDS was completed and controlled for in the analyses. Participants were asked to self-report the development of their secondary sexual characteristics, including growth spurts, body hair growth, skin changes, breast development, and menarche. Except for menarche, which was rated on a two-point scale, all other characteristics were rated using a four-point scale ranging from 1 (not yet started) to 4 (seems completed). The total PDS score was obtained by averaging the point values for all items; higher scores on the PDS are indicative of greater pubertal maturation.

#### 2.2.2. Experimental Pain Tests

##### Temporal Summation Paradigm

Temporal summation measures facilitatory pain processes and may represent the windup phenomenon [39]. Mechanical temporal summation was assessed using a von Frey filament of 6.45 Nm. The pinprick stimuli were applied at a 90° angle to the volar surface of the non-dominant forearm. Ten pinprick stimuli were applied with a frequency of 1/s within an area of 1 cm^2^. Participants rated the pain intensity evoked by a single pinprick stimulus and by a series of 10 identical pinprick stimuli using a mechanical visual analogue scale (VAS). The temporal summation paradigm was repeated twice. The temporal summation value was determined by calculating the difference in pain ratings evoked by a series of 10 stimuli vs. 1 stimulus. The overall temporal summation was determined by calculating the mean values of the two temporal summation tests. A positive value indicates a facilitatory response.

##### Conditioned Pain Modulation (CPM) Paradigms

The CPM paradigms probe endogenous inhibitory pain mechanisms [40]. Based on the recommendations for CPM methodology [41], two CPM paradigms were tested in this study—Heat-CPM and Pressure-CPM. Both paradigms included a control run in which the test stimulus was delivered without the conditioning stimulus. In addition, after an 8 min break, the test stimulus was delivered together with a conditioning stimulus (i.e., the conditioned run). The eight-minute break ensured there was no carry-over effect from the previous test [40].

Heat-CPM control: A continuous 30 s heat test stimulus with an intensity of 46 °C was applied to the volar surface of the non-dominant forearm using a computer-controlled device with a 16 × 16 mm thermode (TSA-2/Pathway, Medoc, Israel). The temperature increase and decrease rate was 6 °C/s from a baseline temperature of 35 °C. Participants rated their pain intensity in response to this test stimulus in real time using a computerized VAS (COVAS, Medoc, Israel). The endpoints of the COVAS were ‘no pain sensation’ and ‘most intense pain imaginable’. The average of heat pain intensity ratings over the 30 s period was calculated.

Pressure-CPM control: A pressure pain threshold (PPT) test stimulus was applied to the upper trapezius muscle on the dominant side using a digital algometer with a circular probe = 1 cm^2^ diameter (Algomed, Medoc, Israel). Pressure was applied at a rate of 60 kPa/s at a 90° angle until participants pressed a button to indicate their first sensation of pain. Four PPT measurements were completed. The first measurement served as a familiarization. The PPT was determined by calculating the mean of the last three measurements.

Conditioning run: Participants immersed their non-dominant foot in a temperature-controlled cold-water bath (Cole-Parmer, USA) set at 8 °C. The conditioning stimulus lasted for 60 s, and the test stimulus (heat or pressure) was applied in the last 30 s of this period. Participants rated the pain intensity from the cold conditioning stimulus using a mechanical VAS at the end of the stimulus. The cold conditioning stimulus was repeated twice (for the heat and pressure CPM response), and the mean cold pain intensity rating was calculated. The order of the heat- and pressure-conditioned runs was quasi-randomized by participant ID number.

The Heat-CPM response was calculated as the difference between heat pain intensity ratings during the conditioning run and the control run, whereas the Pressure-CPM response was calculated as the difference between the PPT obtained during the control run and the conditioning run. A negative CPM value indicates an inhibitory response.

##### Offset Analgesia (OA) Paradigm

The OA paradigm assesses participants’ endogenous inhibitory pain modulation efficiency through mechanisms that are different from CPM [42]. Similar to previous studies assessing OA, the tonic three-temperature stimulus sequence method was employed [42,43]. The first stimulus, T1, had a temperature of 46 °C and lasted 5 s. The second stimulus temperature was T1 + 1 °C, i.e., 47 °C, and lasted 5 s. The third stimulus temperature was at T1 °C, i.e., 46 °C, and lasted 20 s. The T1 heat stimulus had the same temperature as used in the Heat-CPM control run (i.e., 46 °C for 30 s). Like the Heat-CPM control run, the heat stimulus was applied to the volar forearm, and participants rated their pain intensity in real time using COVAS. Similar to a previous study, the OA magnitude was determined by comparing the average pain ratings for the heat stimulus during the time period of 13 to 23 s between the OA paradigm and the Heat-CPM control run [42]. A negative value indicates an inhibitory response.

##### Cold Pain Tolerance

In this test, participants immersed their dominant foot in a cold-water bath set at 8 °C. Participants were asked to immerse their foot until they could no longer hold their foot in the water or until 120 s. The duration for which they kept their foot in the water was recorded as their cold pain tolerance.

### 2.3. Statistical Analysis

Data were collected and stored using Research Electronic Data Capture (REDCap, version 14.0.7). JMP^®^ Pro (Version 16.0.0 SAS Institute Inc., Cary, NC, USA) was used for statistical analyses. Descriptive statistics were generated for demographic data, and the experimental pain sensitivity measures were tested to judge their suitability for parametric analysis. The relationships between experimental pain sensitivity and parental PCS scores were tested with and without controlling factors. To identify the controlling factors, we tested if age and PDS scores are related to experimental pain sensitivity using correlation analysis. Age was not related to any of the measures of experimental pain sensitivity, whereas PDS scores were related to the Heat-CPM response (r = −0.322, *p* = 0.05). Also, PDS and age were significantly correlated (r = 0.678, *p* < 0.0001), and thus, to avoid collinearity, we only controlled for PDS in the regression models. In addition, the parental pain catastrophizing scores were significantly correlated (PCS-Parent and PCS-Parent_child_, r = 0.698, *p* < 0.0001), so separate regression models were performed. Thus, the regression models included either PCS-Parent or PCS-Parent_child_ as independent variables, with PDS and PCS-Child as control factors. The dependent variables were (a) heat pain intensity rating, (b) cold pain intensity rating, (c) cold pain tolerance duration, (d) pressure pain threshold, (e) temporal summation value, (f) OA magnitude, (g) Heat-CPM response, and (h) Pressure-CPM response. To further identify the influence of parents’ psychological profile on their child’s pain, additional exploratory analyses were conducted to identify the differences in experimental pain sensitivity in adolescents with vs. without a family history of psychiatric disorder using unpaired *t*-tests.

A value of *p* < 0.05 (two-tailed) was considered statistically significant. As this is a secondary exploratory analysis of an unpublished study examining the role of sex hormones in experimental pain sensitivity among adolescents, no statistical power calculation was conducted for this analysis. However, our sample size is similar to previous studies that found a significant relationship between pain catastrophizing levels and pain ratings for noxious heat stimuli [10,11].

## 3. Results

Forty-five healthy girls (mean age 12.07 ± 1.47 years) participated in this study. The majority of participating parents were mothers (89%). Descriptive information about the participants is presented in Supplementary Table S1. Surprisingly, the PCS-Child score did not correlate with either the PCS-Parent (r = −0.058, *p* = 0.71) or PCS-Parent_child_ (r = −0.076, *p* = 0.63) scores.

### 3.1. Relationships Between Parental Pain Catastrophizing and Child’s Experimental Pain Sensitivity

**Figure 1:**
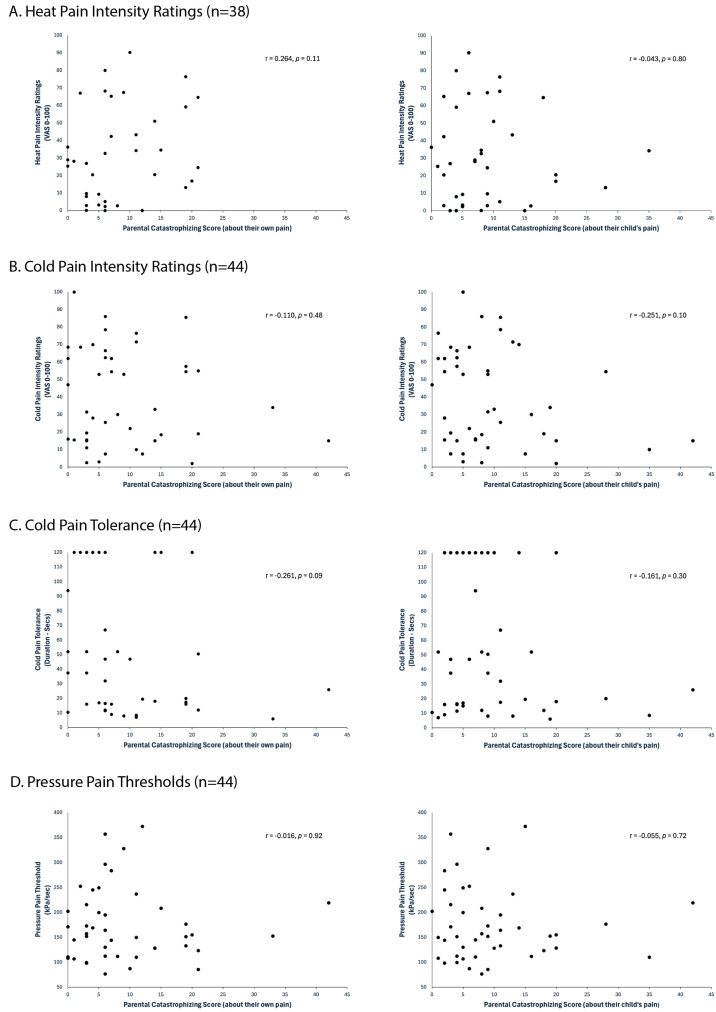

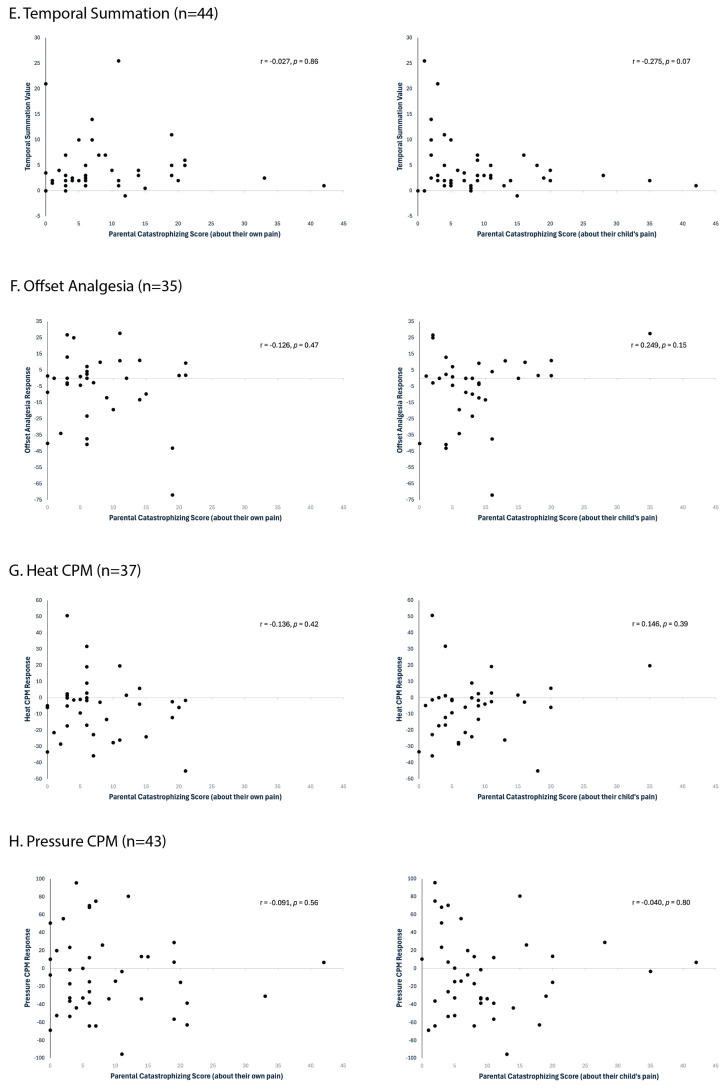
Relationships between experimental pain sensitivity and parental pain catastrophizing about themselves and their children. (**A**) Heat pain intensity ratings. The heat stimulus was 30 s at 46 °C. (**B**) Cold pain intensity ratings. The cold stimulus was 60 s at 8 °C. (**C**) Cold pain tolerance. The cold stimulus was 8 °C and applied for up to 120 s. (**D**) Pressure pain thresholds. (**E**) Temporal summation. Temporal summation was assessed by calculating the difference in pain ratings evoked by a series of 10 pinprick stimuli compared to a single pinprick stimulus. Positive values indicate a facilitatory response. (**F**) Offset analgesia. Offset analgesia was assessed by calculating the difference in pain ratings between the mean pain ratings of 13–23 s of a 46–47–46 °C heat stimulus paradigm and a constant 46 °C heat stimulus. Negative values indicate efficient inhibitory response. (**G**) Heat-conditioned pain modulation (Heat-CPM). Heat CPM was assessed by calculating the difference in mean pain ratings of a heat stimulus (46 °C, 30 s) delivered with and without a conditioning stimulus (8 °C water bath, 60 s). Negative values indicate efficient inhibitory response. (**H**) Pressure-conditioned pain modulation (Pressure-CPM). Pressure CPM was assessed by calculating the difference between pressure pain thresholds without and with a conditioning stimulus (8 °C water bath, 60 s). Negative values indicate efficient inhibitory response.

Even without correction for multiple comparisons, no significant relationships between the parental pain catastrophizing scores (neither PCS-Parent nor PCS-Parent_child_) and the measures of children’s experimental pain sensitivity were found (Figure 1). Controlling for PCS-Child and PDS did not change the results (Table 1). Overall, the amount of variance explained by parental pain catastrophizing (PCS-Parent and PCS-Parent_child_) was low (<17%).

### 3.2. Family History of Psychiatric Disorder

In this study, 27% of the participants (n = 12) had a family history of psychiatric disorders. The most frequently reported disorders were anxiety, depression, and attention deficit hyperactivity disorder (Supplementary Table S1). Even without correction for multiple comparisons, no significant differences in any of the experimental pain measures were found between participants with vs. without a family history of a psychiatric disorder (Table 2). In addition, there were no significant differences in parental and child pain catastrophizing scores between the two groups (Table 2).

## 4. Discussion

This study is the first to comprehensively examine the relationships between parental pain catastrophizing and experimental pain sensitivity in healthy adolescents. Contrary to previous studies reporting relationships between parental pain catastrophizing and clinical pain, and contrary to our hypothesis, we did not observe any relationships between parental pain catastrophizing about themselves (PCS-Parent) or about their child (PCS-Parent_child_) and any measures of experimental pain sensitivity in healthy adolescent girls. Our findings differ from previous studies that demonstrated significant correlations between parental pain catastrophizing and children’s acute and chronic pain intensity/severity [25,26,44,45]. Baseline higher parental pain catastrophizing about themselves is related to greater postsurgical pain intensity in their children after one year [44]. Similarly, higher parental pain catastrophizing about their children is a significant predictor of postsurgical acute pain [26] and is correlated with slower improvement in functional disability scores in children with amplified musculoskeletal pain [45]. However, another study found no association between parental pain catastrophizing about their children and their child’s chronic pain intensity or frequency [46]. The present study assessed experimental pain sensitivity in healthy adolescent girls, which could explain the findings of no significant associations.

Although the PCS can be applied in healthy populations [10,11,47], it is likely that PCS scores are more closely related to clinical pain than experimental pain. The PCS is designed to assess an individual’s psychological experience of pain, capturing cognitive and emotional responses commonly associated with chronic or ongoing clinical pain conditions. Individuals without contextual pain experience might find it challenging to meaningfully respond to certain items of the PCS that reflect the multifaceted experiences of clinical pain. It is plausible that the PCS scores in our study participants do not accurately reflect their pain catastrophizing tendencies in real-life painful conditions. Thus, the relationships between parental pain catastrophizing and children’s pain sensitivity may be more pronounced in adolescents with clinical pain compared to healthy adolescents, who may perceive experimental pain tasks under controlled conditions as less threatening due to the minimal or non-existent risk of harm. Furthermore, compared to parents of healthy children, parents of children with acute/chronic pain tend to be more engaged in their children’s lives through pain-attending solicitous responses and protective behavior, thereby potentially exerting a greater influence on their children’s pain-related beliefs and perceptions, and contributing to pain severity and related disability [27,28,29,30,48]. Moreover, these parents tend to exhibit higher levels of pain catastrophizing, both for themselves and their children, in comparison to parents of healthy children [49]. In our study, the mean (SD) scores of PCS-Parent and PCS-Parent_child_ scores were 9.20 (8.83) and 9.64 (8.86), respectively. These scores are notably lower than those reported in other studies involving children with chronic pain conditions, such as headache, musculoskeletal pain, and functional abdominal pain disorders, where the PCS-Parent_child_ scores ranged from 20.0 to 30.4 [49,50,51]. Thus, the findings of this study may not generalize to parent–child dyads where the child is experiencing acute pain (e.g., post-surgery or trauma) or chronic pain conditions, and higher levels of parental pain catastrophizing may be required to influence a child’s experimental pain sensitivity.

The lack of associations between parental and children’s PCS scores is also surprising. Based on previous studies that demonstrated these relationships [48,50], we assumed that the effect of parental PCS on a child’s experimental pain sensitivity is mediated via the child’s PCS scores. In this context, parental emotions and behavior involving increased catastrophizing impact the child’s cognitive and emotional appraisal of pain, which then leads to increased experimental pain sensitivity. This was suggested as one of the explanations for the relationships observed between parental PCS and children’s clinical pain severity and related interference with daily activities and function [25,28,29,30,45]. However, not only did the present study not find significant associations between parental PCS scores and children’s PCS scores, but it also did not detect relationships between children’s PCS scores and experimental pain sensitivity. This is contrary to previous studies that found relationships between higher levels of pain catastrophizing and increased experimental as well as clinical pain sensitivity [9,10,11,12,13,14].

It is possible that the stimulus modality has a role in the relationships between PCS scores and experimental pain sensitivity. A meta-analysis found overall no associations between psychological measures (anxiety, depression, pain catastrophizing) and CPM responses in healthy controls. However, when paradigms were analyzed by modality, specific associations emerged: anxiety was related to Pressure-CPM, depression to Heat-CPM, and pain catastrophizing to electrical-CPM responses [52]. Thus, the modality of experimental pain measures can influence the outcomes [53,54,55], and it is possible that employing different experimental pain measures or modalities may yield different results.

In an exploratory analysis, no differences in experimental pain sensitivity were detected between adolescent girls with and without a family history of psychiatric disorders. Parental psychiatric disorders, such as anxiety, depression, and post-traumatic stress disorder, adversely affect their children’s development, increasing the likelihood of cognitive-affective and behavioral problems as well as chronification of pain [56,57,58,59]. Moreover, children of parents with psychiatric disorders are more susceptible to developing those disorders, which are also known as comorbidities of chronic pain and may even increase the risk of chronic pain onset during adolescence [58,59,60,61]. Thus, it was hypothesized that adolescents with a family history of psychiatric disorders would exhibit greater experimental pain sensitivity compared to those without such a history. However, no significant differences in experimental pain sensitivity were observed between the groups. This outcome may be attributed to the relatively small number of participants with a family history of psychiatric disorders, which is also the reason why we precluded stratification by the type of disorder or the specific family member with a psychiatric condition. Mothers may exert a greater influence on shaping their child’s pain sensitivity and tend to manifest higher levels of catastrophizing regarding their child’s pain as well as poorer mental health and social functioning compared to fathers [62,63]. Additionally, parents may have a greater negative impact on their child’s pain behavior than siblings. Future studies should examine the effect of PCS scores and the presence of psychiatric disorders in mothers vs. fathers vs. siblings on experimental pain sensitivity in adolescents with and without chronic pain.

### Study Strengths and Limitations

The present study includes a comprehensive assessment of experimental pain, including pain perception and modulation. In addition, two parental pain catastrophizing scales were administered (about themselves and their child). However, a limitation of this study is the relatively small sample size and the inclusion of only healthy girls. Therefore, future research should explore the relationships between parental PCS and experimental pain in healthy boys, as well as adolescents with acute or chronic pain.

## 5. Conclusions

Pain catastrophizing scores of parents of healthy children do not relate to their children’s experimental pain sensitivity. Other biopsychosocial factors may have a greater contribution to the individual variability in experimental pain sensitivity in healthy adolescent girls. Future research should explore these relationships in parents and children with chronic pain.

## Figures and Tables

**Table 1:** Regression models for parental pain catastrophizing and experimental pain sensitivity.

	Estimate (SE)	β	95% CI (Lower, Upper)	*p*
Heat Pain Intensity Ratings, *n* = 38				
Model 1 (R^2^ = 0.070, Cohen’s f^2^ = 0.075)				
PCS-Parent	1.126 (0.71)	0.267	−0.31, 2.56	0.120
PCS-Child	−0.077 (0.49)	−0.026	−1.08, 0.93	0.876
PDS	−0.189 (6.26)	−0.005	−12.91, 12.53	0.976
Model 2 (R^2^ = 0.003, Cohen’s f^2^ = 0.003)				
PCS-Parent_child_	−0.175 (0.62)	−0.049	−1.44, 1.09	0.780
PCS-Child	0.037 (0.51)	0.013	−0.99, 1.07	0.943
PDS	−1.389 (6.54)	−0.037	−14.68, 11.90	0.833
Cold Pain Intensity Ratings, *n* = 44				
Model 1 (R^2^ = 0.127, Cohen’s f^2^ = 0.145)				
PCS-Parent	−0.270 (0.46)	−0.087	−1.20, 0.66	0.562
PCS-Child	0.900 (0.43)	0.311	0.03, 1.77	0.043 *
PDS	4.230 (5.72)	0.110	−7.33, 15.79	0.464
Model 2 (R^2^ = 0.168, Cohen’s f^2^ = 0.202)				
PCS-Parent_child_	−0.686 (0.45)	−0.220	−1.60, 0.23	0.136
PCS-Child	0.870 (0.42)	0.301	0.02, 1.72	0.045 *
PDS	3.680 (5.61)	0.096	−7.64, 15.00	0.515
Cold Pain Tolerance Duration, *n* = 44				
Model 1 (R^2^ = 0.112, Cohen’s f^2^ = 0.126)				
PCS-Parent	−1.423 (0.77)	−0.275	−2.98, 0.14	0.073
PCS-Child	−0.916 (0.72)	−0.191	−2.37, 0.54	0.210
PDS	−4.247 (9.56)	−0.067	−23.56, 15.07	0.660
Model 2 (R^2^ = 0.069, Cohen’s f^2^ = 0.074)				
PCS-Parent_child_	−0.932 (0.79)	−0.181	−2.53, 0.67	0.245
PCS-Child	−0.902 (0.74)	−0.188	−2.39, 0.59	0.228
PDS	−4.536 (9.81)	−0.071	−24.36, 15.29	0.646
Pressure Pain Threshold, *n* = 44				
Model 1 (R^2^ = 0.036, Cohen’s f^2^ = 0.037)				
PCS-Parent	−0.056 (1.28)	−0.007	−2.65, 2.53	0.966
PCS-Child	0.204 (1.19)	0.027	−2.21, 2.61	0.865
PDS	18.431 (15.87)	0.182	−13.64, 50.50	0.252
Model 2 (R^2^ = 0.036, Cohen’s f^2^ = 0.037)				
PCS-Parent_child_	−0.309 (1.28)	−0.038	−2.90, 2.28	0.810
PCS-Child	0.187 (1.19)	0.025	−2.22, 2.60	0.876
PDS	18.155 (15.89)	0.179	−13.97, 50.28	0.260
Temporal Summation, *n* = 44				
Model 1 (R^2^ = 0.082, Cohen’s f^2^ = 0.089)				
PCS-Parent	−0.007 (0.09)	−0.012	−0.19, 0.18	0.939
PCS-Child	0.158 (0.08)	0.287	−0.01, 0.33	0.067
PDS	−0.227 (1.12)	−0.031	−2.48, 2.03	0.840
Model 2 (R^2^ = 0.148, Cohen’s f^2^ = 0.174)				
PCS-Parent_child_	−0.153 (0.09)	−0.259	−0.33, 0.02	0.086
PCS-Child	0.149 (0.08)	0.270	−0.01, 0.31	0.074
PDS	−0.372 (1.08)	−0.051	−2.55, 1.81	0.732
Offset Analgesia, *n* = 35				
Model 1 (R^2^ = 0.088, Cohen’s f^2^ = 0.096)				
PCS-Parent	−0.459 (0.59)	−0.134	−1.67, 0.75	0.443
PCS-Child	−0.230 (0.41)	−0.097	−1.07, 0.61	0.583
PDS	−6.938 (5.11)	−0.237	−17.35, 3.48	0.184
Model 2 (R^2^ = 0.115, Cohen’s f^2^ = 0.130)				
PCS-Parent_child_	0.666 (0.53)	0.213	−0.43, 1.76	0.223
PCS-Child	−0.223 (0.41)	−0.096	−1.06, 0.60	0.578
PDS	−5.765 (5.06)	−0.197	−16.09, 4.56	0.263
Heat CPM, *n* = 37				
Model 1 (R^2^ = 0.132, Cohen’s f^2^ = 0.152)				
PCS-Parent	−0.485 (0.49)	−0.161	−1.48, 0.52	0.330
PCS-Child	0.216 (0.34)	0.104	−0.48, 0.91	0.532
PDS	−8.685 (4.22)	−0.338	−17.27, −0.10	0.048 *
Model 2 (R^2^ = 0.120, Cohen’s f^2^ = 0.136)				
PCS-Parent_child_	0.311 (0.45)	0.114	−0.60, 1.23	0.495
PCS-Child	0.215 (0.34)	0.103	−0.49, 0.92	0.538
PDS	−8.050 (4.28)	−0.313	−16.75, 0.65	0.069
Pressure CPM, *n* = 43				
Model 1 (R^2^ = 0.021, Cohen’s f^2^ = 0.021)				
PCS-Parent	−0.437 (0.80)	−0.086	−2.06, 1.19	0.589
PCS-Child	0.504 (0.75)	0.108	−1.01, 2.02	0.504
PDS	−2.794 (10.12)	−0.044	−23.26, 17.67	0.784
Model 2 (R^2^ = 0.015, Cohen’s f^2^ = 0.015)				
PCS-Parent_child_	−0.189 (0.82)	−0.037	−1.84, 1.46	0.818
PCS-Child	0.515 (0.75)	0.110	−1.00, 2.03	0.497
PDS	−2.887 (10.20)	−0.045	−23.53, 17.75	0.779

PCS-Parent: parental catastrophizing about their own pain; PCS-Parent_child_: parental catastrophizing about their child’s pain; PCS-Child: child catastrophizing about their own pain; PDS: pubertal development scale; CPM: conditioned pain modulation. * *p* < 0.05. *Temporal Summation: higher value indicates facilitatory response. Offset Analgesia: negative value indicates an inhibitory response. Heat- and Pressure-Conditioned Pain Modulation: negative value indicates efficient inhibitory pain modulation response.*

**Table 2:** Experimental pain sensitivity in participants with vs. without family history of a psychiatric disorder.

	Family − Psych (*n* = 32)		Family + Psych (*n* = 12)		*p*
	Mean ± SD	95% CI (Lower, Upper)	Mean ± SD	95% CI (Lower, Upper)	
Pain catastrophizing scores					
PCS-Parent	9.31 ± 1.58	6.13, 12.50	8.92 ± 2.58	3.71, 14.12	0.896
PCS-Parent_child_	9.28 ± 1.58	6.09, 12.47	10.58 ± 2.58	5.37, 15.80	0.669
PCS-Child	14.00 ± 1.84	10.28, 17.72	11.92 ± 3.06	5.75, 18.08	0.563
Experimental pain sensitivity measures					
Heat Pain Intensity	30.63 ± 5.07	20.36, 40.89	30.19 ± 8.08	13.82, 46.57	0.964
Cold Pain Intensity	39.06 ± 4.80	29.38, 48.75	39.87 ± 7.96	23.82, 55.94	0.930
Cold Pain Tolerance	53.45 ± 7.99	37.34, 69.57	45.46 ± 13.25	18.73, 72.18	0.608
Pressure Pain Threshold	172.48 ± 12.65	146.96, 198.0	177.92 ± 20.98	135.6, 220.23	0.825
Temporal Summation	4.32 ± 0.92	2.47, 6.17	4.33 ± 1.52	1.27, 7.40	0.993
Offset Analgesia	−6.52 ± 4.32	−15.30, 2.26	−5.98 ± 6.51	−19.22, 7.25	0.946
Heat CPM	−8.52 ± 3.54	−15.71, −1.33	−1.26 ± 5.55	−12.53, 10.0	0.278
Pressure CPM	−11.23 ± 8.74	−28.88, 6.41	−4.82 ± 14.28	−33.63, 24.0	0.704

Family − Psych: children without a family history (first-degree relative) of psychiatric disorder; Family + Psych: children with a family history (first-degree relative) of psychiatric disorder(s); PCS-Parent: parental catastrophizing about their own pain; PCS-Parent_child_: parental catastrophizing about their child’s pain; PCS-Child: child catastrophizing about their own pain; CPM: conditioned pain modulation.

## Data Availability

Data will be shared upon a reasonable request to the investigators.

## Supplementary Materials

The following supporting information can be downloaded at https://www.mdpi.com/article/10.3390/children11121528/s1: Table S1: Participant characteristics.
