# Supplementary material for: Experimental Pain Sensitivity and Parental Pain Catastrophizing
Source: Children (Basel). 2024 Dec 17;11(12):1528. doi: 10.3390/children11121528 (PMC11726965; doi:10.3390/children11121528)
Supplement: Supplementary file 1 [file children-11-01528-s001.zip › children-3342164-supplementary.pdf]

Supplementary Table S1. Participant characteristics

Age (years)	Mean ± SD	12.07 ± 1.47
	Range	16 - 9
Race	African American	n = 8
	African American, Caucasian	n = 2
	Asian / Pacific Islander	n = 1
	Caucasian	n = 31
	Caucasian, Asian / Pacific Islander	n = 1
	South Asian / Caucasian	n = 2
PCS-Parent Score	Mean ± SD	9.20 ± 8.83
	95% CI (lower, upper)	6.52, 11.89
	Range	42 - 0
PCS-Parent$_{child}$ Score	Mean ± SD	9.64 ± 8.86
	95% CI (lower, upper)	6.94, 12.33
	Range	42 - 0
PCS-Child Score	Mean ± SD	13.44 ± 10.51
	95% CI (lower, upper)	10.29, 16.60
	Range	44 - 3
First Degree Relative with a Psychiatric Disorder Diagnosis (n = 12*)		
Mother	Anxiety, depression, ADHD	n = 5
Father	Depression, ADHD, OCD, bipolar disorder	n = 3
Sibling	Anxiety, depression, ADHD, BPD, PMDD, eating disorder	n = 6

*some families had more than one member with a psychiatric disorder

Abbreviations: PCS-Parent: parental catastrophizing about their own pain; PCS-Parent$_{child}$: parental catastrophizing about their child's pain; PCS-Child: child catastrophizing about their own pain; ADHD = Attention-Deficit/Hyperactivity Disorder; BPD = Borderline Personality Disorder; OCD = Obsessive-Compulsive Disorder; PMDD = Premenstrual Dysphoric Disorder
